# Period Prevalence of Dizziness and Vertigo in Adolescents

**DOI:** 10.1371/journal.pone.0136512

**Published:** 2015-09-11

**Authors:** Thyra Langhagen, Lucia Albers, Florian Heinen, Andreas Straube, Filipp Filippopulos, Mirjam N. Landgraf, Lucia Gerstl, Klaus Jahn, Rüdiger von Kries

**Affiliations:** 1 Department of Paediatric Neurology and Developmental Medicine, Hauner Children’s Hospital, Ludwig-Maximilians-University, Munich, Germany; 2 German Center for Vertigo and Balance Disorders, Ludwig-Maximilians-University, Munich, Germany; 3 Institut of Social Paediatrics and Adolescents Medicine, Division of Epidemiology, Ludwig-Maximilians-University, Munich, Germany; 4 Department of Neurology, Ludwig-Maximilians-University, Munich, Germany; 5 Schön Klinik Bad Aibling, Bad Aibling, Germany; UMR8194, FRANCE

## Abstract

**Objectives:**

To assess the period prevalence and severity of dizziness and vertigo in adolescents.

**Methods:**

In 1661 students in 8^th^-10^th^ grade in twelve grammar schools in Munich, Germany information on vertigo/dizziness was assessed by a questionnaire in the class room setting. Three month prevalence of dizziness/vertigo was estimated; symptoms were categorized as orthostatic dizziness, spinning vertigo, swaying vertigo or unspecified dizziness. Duration of symptoms and impact on daily life activities were assessed.

**Results:**

72.0% (95%-CI = [69.8–74.2]; N = 1196) of the students (mean age 14.5±1.1) reported to suffer from at least one episode of dizziness or vertigo in the last three months. Most adolescents ticked to have symptoms of orthostatic dizziness (52.0%, 95%-CI = [49.5–54.4], N = 863). The period prevalence for the other types of vertigo were spinning vertigo: 11.6%, 95%-CI = [10.1–13.3], N = 193; swaying vertigo: 12.2%, 95%-CI = [10.6–13.8], N = 202; and unspecified dizziness: 15.2%, 95%-CI = [13.5–17.1], N = 253. About 50% of students with spinning vertigo and swaying vertigo also report to have orthostatic dizziness. Most vertigo/dizziness types were confined to less than one minute on average. The proportion of students with any dizziness/vertigo accounting for failure attending school, leisure activities or obliging them to stay in bed were more pronounced for spinning or swaying vertigo.

**Conclusion:**

Dizziness and vertigo in grammar school students appear to be as common as in adults. In face of the high period prevalence and clinical relevance of dizziness/vertigo in adolescents there is a need for prevention strategies. Risk factors for dizziness/vertigo need to be assessed to allow for conception of an intervention programme.

## Introduction

Dizziness and vertigo are common causes of medical consultation in general [1,2]. In adults the life time prevalence of any dizziness type is estimated in 23.2% [3] and the lifetime prevalence of vestibular vertigo bad enough to interfere with activities in 29.5% [1]. The one month prevalence of dizziness varies from 15.8% [4] to 23% [5], and for dizziness severe enough to interfere with normal activities a 10.9% one month prevalence was described [5]. The reported one year prevalence of vertigo was 59.2% for adults [6]. In contrast to adults, epidemiologic data on dizziness and vertigo in children and adolescents are sparse. There are only three population based studies for the age ranges between one and 15 years [7–9] reporting prevalence estimates of dizziness and vertigo: In children aged five to 15 years the one year period prevalence of at least one episode of vertigo or dizziness was 18% and the prevalence for at least three episodes was 5.3% [7]. In another paper the lifetime prevalence of vertigo and dizziness in children aged 1 to 15 years was estimated as 8% [8]. Another study in 10 year old children reported a prevalence of 5.7% [9]. In the first two studies, the wide age range covered is a limitation. The third study mentioned, focused on a particular age, so results cannot be generalized. We are not aware of any data on the period prevalence of dizziness and vertigo for adolescents.

So far assignment to vertigo types cannot be based on an established classification system. Recently, the Bárány Society has launched an effort for a consented classification system [10]. However, until now there is no validate vertigo questionnaires for children and adolescents available.

In this study we assessed the period prevalence of vertigo and dizziness in a large sample of grammar school students (mean age 14.5 ± 1.1). In order to assess the clinical relevance of dizziness and vertigo in these adolescents we also asked for the frequency and duration of the symptoms and their impact on restriction of social activities and school attendance.

## Methods

### Population

The target population for this cross sectional study was grammar school students (aged 12 to 19 years) in Munich. All public grammar schools (academically oriented secondary schools) in the Munich area were invited and principals of 12 out of 47 eligible grammar schools agreed to participate in this study. The reasons for declining participation were its related administrative work load and inability to organize the study in the school setting due to lack of staff. Participants were restricted to students of the 8^th^, 9^th^ and 10^th^ grade. Beyond questions on dizziness and vertigo headache and risk factors of headache were assessed. For the latter condition this cross sectional approach was also used for a cluster-randomized trial [11]. The study was conducted between November 2011 and January 2012. Students with full information about dizziness and vertigo were included for this analysis. The questionnaires were answered during a school lesson and a member of the author team was present to clarify questions.

The study was approved by the Data Safety Officer and the Ethics Committee of the Medical Faculty of the Ludwig-Maximilians-University Munich and the Bavarian Ministry for Teaching and Culture. All parents/guardians and students (> 14 years of age) gave written informed consent to participate in the study.

### Assessment of dizziness and vertigo, vertigo types, duration, frequency and constraints in social activities caused by dizziness or vertigo

Period prevalence of dizziness/vertigo was assessed by the question “Did you suffer from dizziness or vertigo during the last 3 month?” which could be answered with”yes” or “no”. If students ticked “yes” they were asked to further specify the vertigo type: “spinning vertigo like in a carrousel” (spinning vertigo), “swaying vertigo like on a small boat” (swaying vertigo), “feeling of impending black out when rapidly standing up” (orthostatic dizziness), or “neither of the three types” (unspecified dizziness).

For the duration of dizziness/vertigo the following time intervals could be ticked: “up to one minute”, “one minute to 30 minutes”, “30 minutes to four hours”, “four hours to 24 hours”, “one to seven days”, “more than seven days”. In this analysis the last three categories were combined to “more than four hours”.

Students who reported to have experienced dizziness/vertigo in the preceding three months were asked for the frequency of vertigo/dizziness in the last 12 months. The frequencies “once”, “less than five times” or “more than five times” could be ticked.

Constraints in social activities caused by dizziness/vertigo were assessed by the questions “Did the dizziness/vertigo cause considerable constraints in social activities? Did you have to refrain from …” 1) “… attending school because of the dizziness/vertigo?”, 2) “…doing leisure activities because of the dizziness/vertigo?”, 3) “Or did you have to stay in bed because of the dizziness/vertigo?” which all could be answered with “yes” or “no”.

### Statistical methods

Period prevalence for dizziness or vertigo and vertigo types with 95% confidence intervals (CI) were calculated. For the vertigo types a distinction between occurrence of only one type or combination with one, two or three other vertigo-symptoms was made. Furthermore a cross tabulation showing the co-occurrence with other vertigo types was calculated.

For the frequency, duration and constraints in social activities caused by dizziness or vertigo absolute numbers and prevalence with binomial 95%-CI for the four vertigo types were calculated.

## Results

Participation rate was 73.5% (assessed in a random sample of six schools). In total 1661 of the 1674 student participating in this study (mean age 14.5 ± 1.1) of the 8^th^, 9^th^ and 10^th^ grade filled in the questionnaire and gave full information on dizziness/vertigo (<1% drop outs because of incomplete information).

72.0% (95%-CI = [69.8–74.2]) of the students reported to suffer from at least one episode of dizziness or vertigo in the last three month. Most adolescents ticked to have symptoms of orthostatic dizziness (52.0%, 95%-CI = [49.5–54.4]). The period prevalence for the other types of vertigo were between 10–15% (spinning vertigo: 11.6%, 95%-CI = [10.1–13.3]; swaying vertigo: 12.2%, 95%-CI = [10.6–13.8], unspecified dizziness: 15.2%, 95%-CI = [13.5–17.1]. Orthostatic dizziness was more common in girls (58.6%, 95%-CI = [53.3–61.9]) than in boys (44.0%, 95%-CI = [40.4–47.7]; similarly swaying vertigo (girls: 15.0%, 95%-CI = [12.8–17.5]; boys: 8.6%, 95%-CI = [6.7–10.8]) and unspecified dizziness (girls: 17.1%, 95%-CI = [15.3–20.4]; boys: 12.3%, 95%-CI = [10.1–14.9]) whereas there was no significant difference in spinning vertigo (girls: 12.4%, 95%-CI = [10.3–14.7]; boys: 10.7%, 95%-CI = [8.6–13.2]).

**Fig 1:**
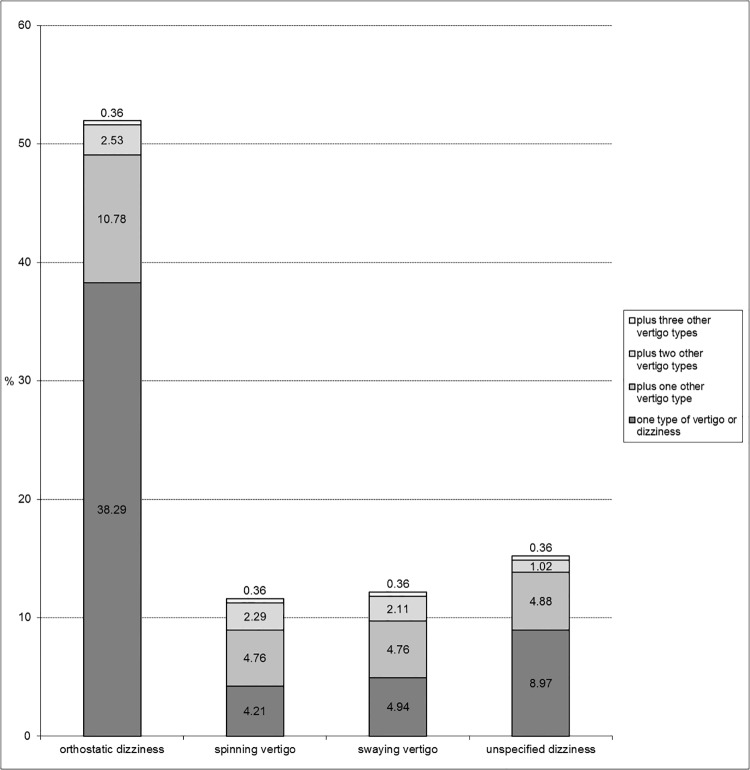
Three-month prevalence vertigo types.

Most students ticked more than one type of vertigo as illustrated in Fig 1. More than half of the adolescents with spinning vertigo and swaying vertigo report to have a combination of vertigo symptoms. Two thirds of the adolescents reporting orthostatic dizziness respectively unspecified dizziness did not report any other vertigo/dizziness type.

In Table 1 vertigo types are cross tabulated one by one. About 50% of students with either spinning vertigo or swaying vertigo also report to have orthostatic dizziness whereas co-occurrence of spinning and swaying vertigo was only observed in 25.9% of adolescents.

**Table 1:** Combination of vertigo types.

	Orthostatic dizziness	Spinning vertigo	Swaying vertigo	Unspecified dizziness
	% (N)			
**Orthostatic dizziness N = 863**	100 (863)	11.47 (99)	11.01 (95)	10.08 (87)
**Spinning vertigo N = 193**	51.30 (99)	100 (193)	25.91 (50)	12.44 (24)
**Swaying vertigo N = 202**	47.03 (95)	24.75 (50)	100 (202)	10.89 (22)
**Unspecified dizziness N = 253**	34.39 (87)	9.49 (24)	8.70 (22)	100 (253)

**Fig 2:**
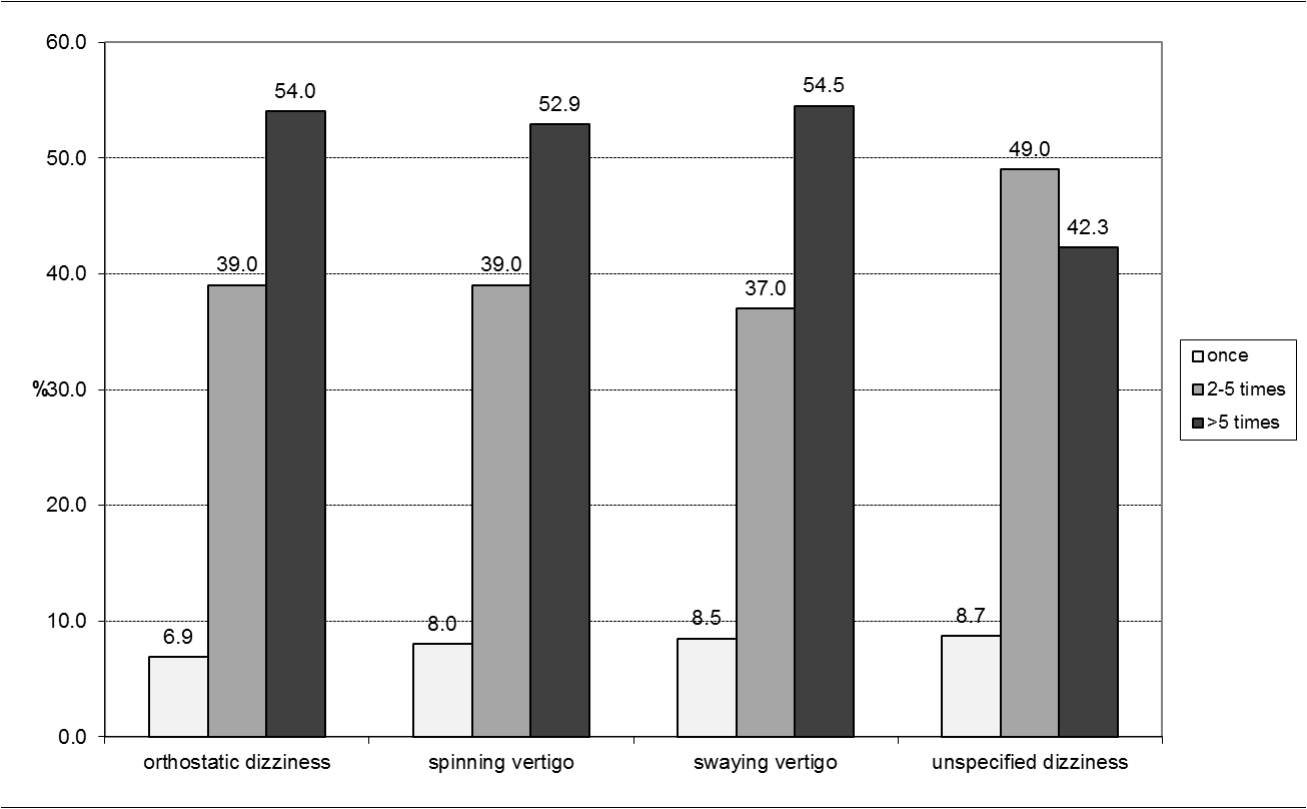
Duration of vertigo types.

Most vertigo/dizziness types were confined to less than one minute on average (Fig 2). Considerable differences with respect to duration between the vertigo types were observed: adolescents with orthostatic dizziness reported shorter duration of less than one minute (64.2%, 95%-CI = [60.9–67.4]) compared to spinning (39.7%, 95%-CI = [32.7–47.0]) and swaying vertigo (40.2%, 95%-CI = [33.3–47.4]), whereas for spinning vertigo and swaying vertigo also durations of 30 minutes up to more than 7 days were reported (spinning vertigo: 17.5%, 95%-CI = [12.3–23.6]; swaying vertigo: 15.6%, 95%-CI = [10.8–21.4]) compared to orthostatic dizziness (6.2%, 95%-CI = [4.6–7.9]) and unspecified dizziness (10.8%, 95%-CI = [7.2–15.3]).

**Fig 3:**
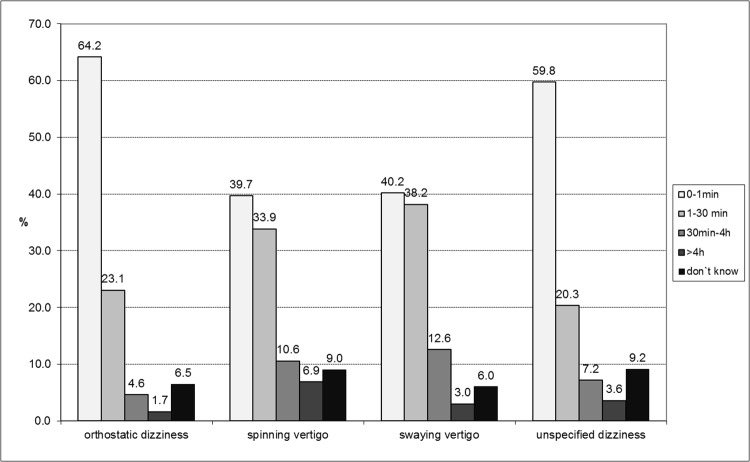
Frequency of vertigo types in the last 12 months.

However frequency of dizziness/vertigo does not differ between the vertigo types (Fig 3). Consistently high frequencies of more than five times were reported by about 50% of the students for every vertigo type except unspecified dizziness.

The proportion of students in whom dizziness/vertigo accounted for failure in attending school or doing leisure activities or obliging them to stay in bed is shown in Table 2. These constraints were more pronounced for spinning vertigo and swaying vertigo. Significant differences compared to orthostatic dizziness were seen for these constraints of not doing leisure activities and not being able of getting up from bed.

**Table 2:** Constraints in social activities caused by dizziness or vertigo.

	**Not attending school**	**Not doing leisure activities**	**Not being able to get up**
	**%95%-CI(N)**		
**Orthostatic dizziness**	9.22[07.35–11.39](77)	18.50[15.92–21.29](115)	19.90[17.25–22.77](167)
**Spinning vertigo**	14.36[09.60–20.34](26)	32.24[25.53–39.53](59)	29.73[23.25–36.88](55)
**Swaying vertigo**	15.98[11.12–21.91](31)	37.19[30.46–44.30](74)	28.93[22.71–35.81](57)
**Unspecified dizziness**	9.20[05.92–13.48](23)	19.52[14.81–24.97](49)	24.19[19.00–30.02](60)

## Discussion

Dizziness and vertigo are common symptoms in adolescents: 72% of adolescents had at least one dizziness/vertigo episode in the last three months. Symptoms described as dizziness are by far more common than those described as vertigo. Attacks of swaying or spinning vertigo are often of higher clinical relevance because of longer duration and impact on social activities compared to attacks of dizziness. In 30% of the cases dizziness and vertigo occurred in the same subject. It is impossible, however, to determine which symptom precedes the other or might be causal.

The high three month period prevalence of orthostatic dizziness, accounting for most of the cases of dizziness/vertigo, contrasts with a much lower prevalence in a population-based study in adults in Germany [12]. In this telephone survey the 12 month prevalence for orthostatic dizziness was 11%. However, the participants were asked for the occurrence of moderate or severe orthostatic dizziness only, whereas we asked in our paper and pencil questionnaire for any orthostatic dizziness regardless the severity. Since orthostatic dizziness had in general only limited impact on social activities in our study, it may be assumed that most students in our study had only mild dizziness. Additionally classification of orthostatic dizziness and vestibular vertigo had to be mutually exclusive in the adult study [12] whereas in our study there was a considerable co-existence between orthostatic dizziness and vertigo. The patient group with combined symptoms of vertigo and dizziness might be substantial, since several vestibular disorders (vestibular migraine, M. Ménière's and benign paroxysmal positional vertigo) are frequently associated with migraine [13–15], and migraine is associated with orthostatic dizziness and syncope [16]. As in adult studies, orthostatic dizziness was more common in girls than in boys [12] and female sex has been identified as an independent risk factor for orthostatic dizziness [17]. The overall prevalence of vertigo—spinning and swaying vertigo in isolation or both combined—was 20.8% in our study and in the same range as in most studies in adults [1–6]. The lower prevalence in the Neuhauser study [1] is likely to be a reflection of confinement to moderate and severe symptoms and exclusion of combined symptoms. The prevalence estimates in our study are higher than in previous studies in children [7–9] suggesting that the prevalence in adolescents is more similar to the prevalence in adults [1–6]. This is an important finding since there are no other studies addressing the prevalence of vertigo/dizziness in adolescents specifically.

An interesting finding is the co-occurrence of different types of vertigo, which has been also noticed by Humphriss et al., who found 26.8% of the children (all aged 10 years) reporting at least 3 different descriptions of their dizziness [9]. Dizziness, swaying and spinning vertigo have been reported to be associated with migraine [13,15]. The diagnostic entity of vestibular migraine [18] has reached international acceptance. There are other clusters of vertigo/dizziness symptoms such as the association of vestibular vertigo syndromes and vestibular migraine to somatoform dizziness/vertigo [19,20]. Co-occurrence of these symptoms may reflect the described co-occurrence of different vertigo syndromes. An alternative explanation might be the subjective nature of the clinical symptoms, which is difficult to assign to a specific category. Therefore students might have ticked a combination of different descriptions.

Another important finding is the high impact of vertigo symptoms on restriction of social activities pointing to the high clinical relevance of vertigo. Similarly in the survey of Neuhauser et al. [21], which only ascertained moderate and severe vertigo, the reported dizziness/vertigo was severely impairing in almost two-thirds of cases, leading to sick leave, medical consultation or interruption of daily activities. Niemensivu reported vertigo severe enough to stop the present activity in 69% of affected children aged 5 to 15 years [8]. Applying the same case definition Humphriss and Hall found that 51.5% of the 10 year old children suffering from vertigo had to stop their present activity [9]. In our study a more explicit definition of interference in chosen: we assessed inability to go to school, participate in leisure activities or even were forced to lie down. Surprisingly even 10% of students with orthostatic dizziness—which could be assumed as less impairing—reported to not attend school because of their dizziness. A possible explanation for this could be the coexistence with other types of vertigo. However, nearly half of those students who reported not attending school suffered from isolated orthostatic dizziness. Unspecified dizziness, which can also include non-organic vertigo or dizziness, was observed in only 8% of those students.

The strength of the study is the focus on adolescents, an age group previously insufficiently investigated. Most students (98%) participating in this study are in the age range of 13 to 17 years. To our knowledge this is the first study ascertaining the period prevalence of vertigo in an adolescent population of up to 19 years and examining its clinical characteristics and its consequences on daily and school activities.

One limitation of the study is the restriction of the study population to students from grammar schools—about 50% of the birth cohort [22]—which may limit external validity: children in grammar schools are more likely to come from socially advantaged families with higher income. Whether this results in an over-estimate of prevalence (e.g. from over-attentive parents or more articulate parents/children) or underestimates the prevalence of vertigo/dizziness in the general population (e.g. if vertigo/dizziness is socially patterned like other health outcomes and a higher socioeconomic status would be associated with better health states) remains unclear.

Furthermore, all results are based on self-administered questionnaire by the students which has to be considered in the comparison to other studies.

Participation rate was 73.5%. The 26.6% of the student not participating in our study could be explained by 1) students/parents gave no consent to participate or 2) students did not attend class on that day. However, selection bias due to these reasons is rather unlikely: 1) It may be possible that children/parents were more likely to consent if they had a problem with headaches, which might have resulted in a higher estimate of prevalence if dizziness is associated with headache (as it is in migraine). However, headache prevalence in this study sample (84%) is comparable to many other studies in this age group [11]. 2) Possible students did not attend class because they had headache or vertigo resulting in an underestimation of vertigo. This is very unlikely as we found a very high vertigo prevalence.

A limitation of all studies on vertigo/dizziness is the lack of an established international classification for dizziness/vertigo as it is available for other subjective complaints such as headache (for example the ICHD for headache [23]). Recently, the Bárány Society has launched an effort for a consented classification system [10]. So far assignment to vertigo types cannot be based on an established classification system making it difficult to validate vertigo questionnaires for children and adolescents. Since neither the German nor the English language differentiate very clearly between dizziness and vertigo we presented our questions on dizziness/vertigo in a simple and coherent way with examples for the respective vertigo types. The "vocabulary" problem could also be a probable contributor to the many instances of "double-ticking" for different types of vertigo.

Furthermore vertigo type specific prevalence estimates show large confidence intervals showing imprecision, which is a reflection of the small sample of students with spinning, swaying vertigo. Despite the small sample sizes and possible uncertainties as how to label the vertigo types, however, significant differences between dizziness and both spinning and swaying vertigo are observed.

## Conclusion

Period prevalence dizziness and vertigo in grammar school students has already reached adult levels. Although we could not assess whether this also pertains to adolescents in other school types, this high period prevalence and clinical relevance of dizziness/vertigo points to a need for prevention strategies.

## Supporting Information

S1 Dataset (CSV)
